# Eating Attitudes, Stress, Anxiety, and Depression in Dietetic Students and Association with Body Mass Index and Body Fat Percent: A Cross-Sectional Study

**DOI:** 10.3390/diseases12050108

**Published:** 2024-05-20

**Authors:** Olga Magni, Paraskevi Detopoulou, Evangelia Fappa, Anastasia Perrea, Despoina Levidi, Vasilios Dedes, Milia Tzoutzou, Aristea Gioxari, Georgios Panoutsopoulos

**Affiliations:** 1 Department of Nutritional Sciences and Dietetics, University of the Peloponnese, 24100 Kalamata, Greece; 2 Department of Clinical Nutrition, General Hospital Korgialenio Benakio, 11526 Athens, Greece

**Keywords:** disordered eating, anxiety, body mass index, university students

## Abstract

University students face challenges impacting psychology and dietary choices. The present work examined the association between eating attitudes, stress, anxiety, and depression to body mass index (BMI) and body fat percentage in Dietetics students. Respondents completed the Eating Attitudes Test-26 (EAT-26), the Depression Anxiety Stress Scales (DASS), a validated Food Frequency Questionnaire, and the Hellenic Physical Activity Questionnaire (HPAQ). Anthropometry and a bioimpedance analysis were performed. The EAT-26 score was 11 (8–16) and the DASS score was 20 (11–36) (medians and interquartile ranges). Disordered eating was detected in 20% of men and 15% of women. Depressive symptomatology was detected in 30% of males and 23% of females, anxiety in 35% of males and 40% of females, and stress in 29% of males and 35% of females. EAT-26 and DASS scores were highly correlated (r = 0. 0.221, *p* = 0.001). The EAT-26 oral control subscale (B = 0.430, SE = 0.184, *p* = 0.026) was positively correlated with BMI in men in the models, adjusted for age, physical activity, and Mediterranean Diet Score, while no association was documented for % body fat. The DASS depression score was not related to BMI in multi-adjusted models. In conclusion, disordered eating, depression, stress, and anxiety are present in this sample of university students. The relationship between disordered eating and BMI needs consideration in programs targeting overweight or underweight in Dietetics students.

## 1. Introduction

Entering higher education is a transitional period, accompanied by significant changes and often a move away from the parental shelter [1]. Students adopt new social roles and make important decisions concerning their lives and careers [2]. Several challenges may be present, such as seeking employment for the first time, limited leisure time, peer pressure, the need to excel, living with other people, not meeting parental expectations, homesickness, certain social problems, and sleep disorders [3,4,5]. The changes in an individual’s life may affect dietary choices and eating habits [6]. In addition, the aforementioned conditions may adversely affect the mental health of some students, causing insecurity which, over time, may contribute to the appearance of anxiety, depression, eating disorders, and other related conditions [2]. It is also noted that eating attitudes may differentiate or more intensively change in Dietetic students [7], since they have greater nutrition knowledge [8,9].

Eating disorders are classified as psychiatric conditions characterized by deviant eating or weight control attitudes [10]. Τhey include anorexia nervosa, bulimia nervosa, and binge eating disorder, as outlined in the Diagnostic and Statistical Manual of Mental Disorders Fifth Edition [11]. The terms “disordered eating” or “disordered eating behavior” do not refer to a clinical diagnosis, as they do not meet the criteria described in DSM-5, primarily due to differences in the frequency and severity of the symptoms [10,11]. They include a negative attitude regarding weight and shape, abnormal eating behavior, and unhealthy weight control behaviors [10,12].

Depression is a heterogenous constellation of symptoms. Several subtypes of depression can be found according to the presenting symptoms. Lately, two main subtypes have been proposed: type 1, characterized by reduced appetite, weight loss, sleeping difficulties, and suicidal thoughts; and type 2 (atypical), presenting with augmented appetite, increased weight, a sense of heaviness in limbs, hypersomnia, and a dysmetabolic profile [13].

Several observational studies have examined the correlations between weight status and diet, eating attitudes, stress, anxiety, and depression, with mixed outcomes. In some studies, no significant associations were found between eating attitudes and body mass index (BMI) [14,15], while, in others, a positive correlation between BMI and disordered eating was documented [16,17]. Moreover, disordered eating has been associated with other adiposity measures, i.e., waist circumference, waist-circumference-to-hip-circumference ratio, and body fat percentage [17]. Regarding stress, some cross-sectional studies have identified an association between elevated BMI and anxiety–stress levels [18,19] with possible sex-related differentiations [20], while others have not found any significant associations [5,21]. A single study identified a correlation between stress and both weight gain and weight loss [22]. Concerning the correlation between depression and body weight status, the associations were not significant in several studies [5,18,21].

Researchers in Greece have already addressed the association between psychological status, disordered eating, and body weight, with conflicting results. In particular, a positive association has been found between a higher body weight and disordered eating [23,24]. Stress and anxiety have both been positively [25] and negatively associated to body weight [26]. However, the aforementioned evidence pertains to the pre-COVID-19 outbreak period. Notably, in the COVID-19 era, studies have suggested high rates of depression, anxiety, and stress [27,28,29]. However, the extent to which these conditions are related to body weight and how the COVID-19 pandemic may have impacted these issues is not clear.

Thus, the current work aimed to assess the association of eating attitudes, stress, anxiety, and depression to adiposity measures (i.e., BMI and % body fat) in university students during COVID-19, with a special focus on Dietetics students. 

## 2. Materials and Methods

### 2.1. Sample

Undergraduate students (*n* = 143) from the Nutritional Sciences and Dietetics Department of a University participated in the study. Students were notified electronically by the secretariat of the Department of Nutrition and Dietetics. Advertising materials were displayed in areas of the university frequented by students, such as the cafeteria and the restaurant.

### 2.2. Ethics

The School of Health Sciences Research Ethics Committee at the University of Peloponnese has given approval to conduct the study (protocol number 129, 7 June 2022). All the included procedures were in line with the ethical standards of the University’s committee, as well as the Declaration of Helsinki (1964 and subsequent modifications). All respondents signed a consent form for their participation in the study.

### 2.3. Questionnaires

#### 2.3.1. General Information

Data regarding general information for the respondents were collected through a 12-item general information self-completed questionnaire. It included questions on age, sex, university department, and year of study. Data regarding smoking, and family medical history were also collected. More particularly, smokers were classified as current smokers, non-smokers, and former smokers. For current smokers, the daily quantity of cigarettes was recorded, as well as the sum of smoking years. The family history for hypercholesterolemia, hypertension, obesity, and type 2 diabetes was recorded (yes or no).

#### 2.3.2. Assessment of Eating Attitudes

Students’ eating attitudes were assessed with the Eating Attitudes Test 26 (EAT-26) questionnaire [30]. EAT-26 is a self-administered shortened and revised form of the 40-item Eating Attitudes Test questionnaire [31]. It is a widely used tool for assessing attitudes, emotions, and concerns related to eating, body weight, and exercise and has been shown to have good psychometric properties, also for college students [15,16,24]. Furthermore, it contributes to the identification of eating attitudes related to disordered eating [14,17]. It consists of 26 items, each of which is rated on a 6-point Likert scale, and are clustered into three subcategories: (i) the dieting, (ii) the bulimia and food preoccupation, and (iii) the oral control subscale [16,17,32]. The dieting subscale refers to deliberate avoidance of high-calorie foods, preoccupation with weight loss, and concerns about body image, shape, and size [30]. It includes 13 questions and ranges from 0 to 39. Regarding bulimia and food preoccupation, statements refer to food-related thoughts that may indicate psychogenic bulimia [30]. The bulimia subcategory includes 6 questions and its score ranges from 0 to 18. The subscale of oral control is connected to self-regulating eating habits alongside the perceived weight gain pressure from external individuals [30]. The oral control subcategory includes 7 questions and its score ranges from 0 to 21. The total score is calculated by summing all questions’ grades, and ranges from 0 to 78 [14,17]. If the score reaches at least 20 points, the examinee is considered to be at high risk of developing an eating disorder [14,15,17,24,31].

#### 2.3.3. Assessment of Depression, Anxiety, and Stress

To examine the presence of depression, anxiety, and stress symptoms, the Depression Anxiety Stress Scales (DASS) questionnaire [33] was administered, appropriately modified for the population studied [34]. Respondents were requested to rate on a 4-point Likert severity/frequency scale the extent to which each sentence corresponded to how they had felt the previous week (0 = did not apply to me at all, 1 = applied to me to some degree, or some of the time, 2 = applied to me to a considerable degree, or a good part of time, and 3 = applied to me very much, or most of the time [33,34]. It consists of 42 items related to Depression (*n* = 14 items), Anxiety (*n* = 14 items), and Stress (*n* = 14 items) [33]. For the Depression scale, a score of 0–9 is considered normal, a score of 10–13 is considered mild, a score of 14–20 is considered moderate, a score of 21–27 is considered severe, and a score greater than 28 is considered extremely severe. For the Anxiety scale, a score of 0–7 is considered normal, a score of 8–9 is considered mild, a score of 10–14 is considered moderate, a score of 15–19 is considered severe, and a score greater than 20 is considered extremely severe. For the Stress scale, a score of 0–14 is considered normal, a score of 15–18 is considered mild, a score of 19–25 is considered moderate, a score 26–33 of is considered severe, and a score greater than 34 is considered extremely severe. The Depression scale refers to distress, hopelessness, underestimation of life and self, lack of interest and involvement, anhedonia, and apathy/inactivity [33]. In parallel, the Anxiety scale refers to the physical symptoms of anxiety: the autonomic nervous system arousal, the impact of anxiety on skeletal muscles, anxiety related to specific scenarios, and the subjective experience of anxiety’s effects [33]. The Stress scale measures difficulty in relaxation, overstimulation of the nervous system, agitation, irritability/over-reactivity, and impatience [33]. The score on each scale is calculated by summing the grades of each one of the relevant 14 items. The overall score is obtained by adding the 3 scales’ sub-scores [33].

#### 2.3.4. Assessment of Dietary Habits and Diet Quality

A valid semi-quantitative Food Frequency Questionnaire (FFQ) was administered [35]. The FFQ included 69 foods and beverages. Moreover, based on respondents’ responses to the FFQ, the Mediterranean Diet Score (MedDietScore) was calculated ranging from 0 to 55 [36]. Higher scores represented higher adoption of the Mediterranean Diet [36].

#### 2.3.5. Assessment of Physical Activity

A self-administered physical activity questionnaire, developed and validated for the present population, was administered (Hellenic Physical Activity Questionnaire, HPAQ) [37]. The Metabolic equivalents (METs) were calculated in MET/min [37]. One MET/min is the energy consumed during a minute of resting (MET = 1). Higher MET/min scores are related to higher levels of physical activity.

### 2.4. Anthropometry and Body Composition

Body weight was measured to the nearest 0.1 kg (Tanita MC-780, TANITA, Tokyo, Japan), and height was measured to the nearest 0.1 cm with a stadiometer (Seca, Hamburg, Germany) [38]. BMI was calculated by the division of weight (kg) to height squared (m^2^) [39].

For waist circumference, a tape measure was placed around the narrowest part of the waist, i.e., between the last rib and above the level of the navel, whereas, for hip circumference, the tape measure was placed at a level ensuring the maximum hip circumference [40,41]. Wrist circumference was also measured. All circumferences were measured to the nearest 0.1 cm (Seca, Hamburg, Germany) at a standing position [40].

The thickness of different skinfolds was measured: biceps, triceps, subscapular, and suprailiac, according to standard procedures [42]. Measurements were performed with a caliper on the right body side (Slim Guide Caliper, HaB Essentials). Measurements were recorded to the nearest 0.2 mm [42]. The measurements were performed three times at each site and the average value was used. After identifying the midpoint of the arm length, which is between the acromion and the olecranon, vertical folds were made to measure the biceps and triceps skinfolds [42]. The anatomical site for subscapular skinfold measurement was located by palpation of the area. This was followed by a diagonal measurement 1 cm below the lower angle of the scapula [42]. The suprailiac skinfold was measured above the iliac crest in the mid-axillary line [42].

For body composition analysis, the bioelectrical impedance analysis (BIA) method was applied using a device (Tanita MC-780, Japan) that provides data for % body fat, phase angle, trunk and limb muscle mass, and intracellular and extracellular water [43]. Participants abstained from engaging in strenuous physical activity, consuming alcoholic beverages, and consuming high-caffeine drinks for at least 8 h before the BIA measurements [42]. They also refrained from eating and drinking for at least 3 h before the designated measurement time [42]. To avoid possible fluctuations in water retention, measurements were not taken during nor within 3 days before or following menstruation in women. All participants were in an upright standing posture throughout the measurements. Shoes and socks were taken off, and the weight of the clothes was recorded. All measurements were performed by two members of the technical staff of the Department of Nutritional Sciences and Dietetics.

### 2.5. Statistical Analysis

To test whether variables were normally distributed, the Kolmogorov–Smirnov test was used. Means and standard deviations are presented for continuous normally distributed variables, while medians and interquartile ranges (IQRs) (25th–75th percentile) are presented for those not normally distributed. Qualitative categorical variables are presented as absolute values. The chi-square criterion was performed for comparisons between categorical variables. Comparisons of continuous variables were conducted with the t-test for variables with normal distribution, and the Mann–Whitney test for those non-normally distributed.

Bivariate correlations between parameters were performed using Spearman’s correlation coefficient in order to also capture non-linear associations. The relationship between eating attitudes, depression, anxiety, stress, and body weight status was examined using multivariate linear regression models. BMI and % body fat (both variables met the normality criterion) were used as dependent variables. Age, gender, physical activity, dietary factors, and EAT-26 or DASS scores, as well as their subcategories, were used as independent variables. The statistics were performed with IBM SPSS Statistics Inc. (version 29.0, Chicago, IL, USA).

## 3. Results

### 3.1. Demographic, Anthropometric, and Psychological Characteristics of the Respondents

The analysis consisted of 143 respondents, comprising 108 (75.5%) females and 35 males (mean age 21 years). The total number of enrolled Dietetics students is 405 students, 96 males and 309 females. This means that 35.3% of the students responded (36.5% of males and 35% of females, with similar respondent rates). The percentage of current smokers was 19.6%. In Table 1, the demographic and anthropometric data of the respondents are shown. The average BMI of the study sample was 22.0 kg/m^2^ (females—22.8 kg/m^2^ and males—21.7 kg/m^2^). It is noted that overweight and obese subjects were grouped, due to the low frequency of obese subjects. We found that 89out of 108 female students (82.4%) were classified as having a normal BMI, 13 (12%) were underweight, and 12 (11.1%) were overweight or had obesity; 26 out of 35 male students (74.2%) had a normal BMI, 2 (5.7%) were underweight, and 9 (25.7%) were overweight or had obesity.

In the total sample, the median total score for EAT-26 was 11 (IQR: 8–16), while the median scores for the EAT-26 dieting scale, bulimia scale and oral control scale were 7 (IQR: 4–10), 2 (IQR: 1–4), and 1 (IQR: 0–3), correspondingly. Among women, 15% scored over 20 on the EAT-26, indicating disordered eating, compared with 20% of men.

In the total sample, the DASS score was 20 (11–36) (median, IQR). The scores for the depression, anxiety, and stress subcategories were 4 (1–9.2), 5 (2–10), and 11 (6–17), correspondingly (medians, IQR). Almost 22.9% of women and 30.3% of men experienced depression, while anxiety affected almost 39.7% of women and 35.3% of men. Stress was reported by 34.6% of female and 29.4% of male respondents. 

Furthermore, in Table 1, the demographic and anthropometric characteristics of the respondents are shown in relation to the EAT-26 scores. The suprailiac skinfold median between subjects with EAT-26 > 20 (8.3) and EAT-26 < 20 (10.6) scores demonstrated a significant difference (*p* = 0.008). In individuals with an EAT-26 > 20 score, the triceps skinfold median was significantly (*p* = 0.029) lower (13.2) compared to those with EAT-26 < 20 scores (15.0). Similarly, the median of the sum of skinfolds in individuals with EAT-26 > 20 scores (36.9) was significantly (*p* = 0.027) lower compared to those with EAT-26 < 20 scores (46.8).

It is noted that not all students answered the DASS questionnaire; that is why the demographic and anthropometric characteristics of respondents are also shown in relation to the DASS scores (Supplementary Table S1). There was no differentiation of DASS scores according to the year of study, and measures of anthropometry or body composition.

The EAT-26 score and its subcategories showed a positive association with the DASS score and its subcategories in the whole sample, as shown in Table 2. After sample stratification by gender, significant correlations were found solely in female respondents.

### 3.2. Bivariate Correlations between Eating Attitudes, Depression, Anxiety, and Stress, and Body Composition

Bivariate correlations between the EAT-26 score and BMI, and % body fat, as well as other body composition variables, are shown in Supplementary Tables S2–S4 for the total sample, women, and men, correspondingly. Specifically, in the total sample, a correlation was found between the EAT-26 diet and waist circumference (r = −0.235, *p* = 0.005), visceral fat (r = −0.168, *p* = 0.045), and extracellular water (r = −0.244, *p* = 0.003), indicating possible dehydration. A negative association was documented between disordered eating and BMI (r = −0.162, *p* = 0.050), waist circumference (r = −0.235, *p* = 0.005), and body fat mass (measured in kilograms) (r = −0.162, *p* = 0.053). Moreover, the EAT-26 oral control subscale score was positively correlated with BMI (r = 0.178, *p* = 0.031) in the total sample. Among female students, a negative correlation was observed between the total score of EAT-26 and extracellular water (r = −0.196, *p* = 0.044). The diet subscale score of EAT-26 was inversely correlated with waist circumference (r = −0.228, *p* = 0.018), and extracellular water (r = −0.303, *p* = 0.002). These negative correlations were also observed in male respondents. The subcategory score for the EAT-26 diet showed a negative correlation with % body fat (r = −0.347, *p* = 0.038) and body fat in kilograms (r = −0.329, *p* = 0.050).

Negative significant correlations existed between the anxiety-related score and BMI (r = −0.185, *p* = 0.026), waist circumference (r = −0.185, *p* = 0.029), and extracellular water (r = −0.177, *p* = 0.035). In women, a negative correlation was found between anxiety and waist circumference (r = −0.189, *p* = 0.050).

### 3.3. Multivariate Linear Regression Models Regarding the Association of Eating Attitudes and Depression, Anxiety, and Stress with BMI

Linear regression models were applied to explore the possible association between the factors studied. A linear regression model was established, with BMI as the dependent variable (Table 3). The independent variables entered in the models were as follows: age, physical activity, and MedDietScore and ΕAΤ-26 and DASS scores (total and subcategories’ scores). The EAT-26 oral control subscale was positively associated with BMI in men (Table 3) and the association was significant in all three models. No significant association was found between the BMI and EAT-26 total scores, as well as with the EAT-26 dieting and bulimia subscales, neither in men (Table 3) nor in women (Supplementary Table S5). The DASS scores and their subscales’ scores were also not significantly associated with BMI, neither in men (Table 3) nor in women.

### 3.4. Multivariate Linear Regression Models Regarding the Association of Eating Attitudes and Depression, Anxiety, and Stress with % Body Fat

Linear regression models were developed with % body fat as the dependent variable and age, physical activity, and EAT-26 score or DASS score as independent variables. No significant association was found between EAT-26 and % body fat in the whole sample (B = −0.082, std. error = 0.087, *p* = 0.348), men (B = −0.0004, std. error = 0.145, *p* = 0.998), and women (B = −0.036, std. error = 0.087, *p* = 0.586). No statistically significant correlation was found between the DASS score and % body fat in the whole sample (B = −0.002, std. error = 0.034, *p* = 0.947), men (B = −0.023, std. error = 0.061, *p* = 0.707), and women (B = −0.015, std. error = 0.031, *p* = 0.620). Similarly, no associations were documented regarding the EAT-26 and DASS score subscales, when they were used in the models as independent variables.

## 4. Discussion

The current study uncovered a small yet noteworthy proportion of Dietetics students with disordered eating. Additionally, fewer than half of them displayed signs of depression, anxiety, or stress. Male students with higher EAT-26 oral control scores had a higher BMI.

Comparing the present findings with those of similar works, it was observed that the respondents’ median total EAT-26 score was lower than the median or mean of adults in Turkey (mean: 15.0) [14], as well as that of similar-age women living in Argentina (mean: 18.34) [44]. However, the median score of EAT-26 in the present study was higher compared to that reported for Health Science students in Iran (median: 7.0) [17]. Similar results to our study were found in a previous study of high school students in Greece [24]. The proportion of students with an EAT-26 score of >20 is higher than the corresponding rates found in Health Science students from India (7.8%) [16] and from the United States of America (10%) [15]. Therefore, an increased risk of disordered eating is evident when compared to findings from previous similar studies. This may be explained by the fact that the subjects of the current study were Dietetics students. Indeed, a recent review indicated that 4–32% of students in Dietetics are at a high risk of eating disorders [45]. Several tools have been used to evaluate the risk of disordered eating [45]. Two studies [46,47] used the original EAT-40 (40 questions), four studies used EAT-26 [48,49,50,51], while other tools were also used, such as the modified Yale food addiction scale and the Munich Composite International Diagnostics Interview [45].

The relative increased risk of disordered eating in Dietetics students has both personal and societal components to consider. For example, perfectionism and insecurity have been reported by Dietetics students [47]. The word “dietitian” on the web has been also connected to images of “thin, young, pretty, white, and female” individuals, as recently suggested [52]. In parallel, many dietitians are a priori interested in the field of nutrition to pursue a degree in it. Consequently, it is conceivable that the behaviors and attitudes observed in individuals with eating disorders might also occur among nutrition students. However, it is crucial to emphasize that this does not necessarily suggest that Nutrition students have disordered eating themselves. Moreover, as recently reviewed, most studies investigating disordered eating in Dietetics students have been mostly conducted on female students [45]. However, the present study revealed that disordered eating is also a concern for male students.

The levels of depression, anxiety, and stress reported by students in the current sample were lower than those reported by students in Egypt [18] and higher than those reported by students in Spain [5]. Greek students have, also, previously been the subject of research regarding anxiety and depression. Specifically, findings from previous studies have shown that students at the University of Patras, as well as those of other Greek universities, expressed similar percentages of anxiety to ours (35.8% and 36.8%, respectively) [29,53]. Higher levels of stress have been reported for students in Greek universities (40.3–47.3%) [27,53], while the highest anxiety levels have been reported for Greek medical students (67.6%) [54]. Regarding depression, higher rates have been reported in students from Patras University (51.2%) [29], Aristotle University of Thessaloniki (55%) [27], and five Greek universities (44.8%) [53]. A survey of medical students in Greece found very high levels of depression reaching 74.3%, although other tools were used for assessment [54]. However, similar rates of depression to the present study were identified in Greek female nursing students during COVID-19 [28]. Regarding depression, anxiety, and stress, in a study of ~600 Dietetics students, a similar percentage to our students had high scores on DASS [55]: 30% of them experienced depression, 40% of them had anxiety, and 27% experienced stress [55]. These results are similar to our findings.

As far as Dietetics students are concerned, it has been shown that no difference exists in EAT-26 scores of Dietetics students and those of other specialties [56]. Indeed, when Matusik et al. corrected for BMI, the risk of eating disorders was higher among students of other majors rather than those studying Dietetics. Another study indicated that 14% of those studying Nutrition and Dietetics presented disordered eating (EAT-26 greater than 20) [9], which is lower than our results. In the same study, disordered eating was linked to the year of study, as 24% of first-year students had a score of ≥20 compared to 9% of students enrolled in later years [9]. In this sample the presence of disordered eating was not related to the year of study (*p* = 0.358). There was no association found between the EAT-26 scores and gender or degree of enrolment [9]. Additionally, a study in dietetics students in Chile showed that 23.3% of the examined population were susceptible to developing orthorexia nervosa, in which an obsession with healthy eating habits is present [57]. Among 176 dietetics students in Greece, 68.2% presented with orthorexia, whose symptoms were associated with an increased BMI and waist circumference [58].

It is noted that, in the present study, a positive association between the EAT-26 and DASS score was documented, implying that students with a higher susceptibility to disordered eating had a greater severity of depression, anxiety, and stress. A study carried out among Pakistani university students also illustrated a positive correlation between the EAT-26 and DASS-21 scores [59]. The EAT-26 scale indicated a significant association with stress, anxiety, and depression. Middle school students in China with eating disorders were more prone to experiencing stress, anxiety, and depression than their classmates without eating disorders [60].

In terms of body weight status, the mean BMI of the study’s population was 22.0 kg/m^2^, with 76% of students falling within a normal BMI range. Similar to the present study, findings on body weight status have been published for students from Greece [61], Spain [5], Iran [17], and India [20]. It is of interest, also, that, in some cases, female students seem to be either thinner or more likely normal-weighted than male ones [62], as also supported by findings in India where over one-third of the female respondents had a BMI below normal values (normal BMI: 49.4%, underweight: 36.5%, overweight: 7.4%, and obesity: 2%) [16]. In the US, one study reported similar results to ours [15]. 

The present study’s findings regarding the positive association between disordered eating and BMI are in line with previous research [14,16,17,63]. In those studies, students from Turkey, Africa, India, and Iran were included, and similar associations were found [16,17,63]. In parallel, a positive association has been reported between the BMI and EAT-26 dieting subscore, while a negative association has been documented between the BMI and EAT-26 oral control subscore [24], in contrast to our results. In other studies, no significant associations were found between eating attitudes and BMI [14,15]. Interestingly, in the present study, the % body fat was not related to EAT-26, while the suprailiac and triceps skinfolds thicknesses were lower in the students with high EAT-26 scores. Additional research has shown body fat, as assessed by the skinfolds sum, is related to EAT-26 scores [64]. It is noted that, among the anthropometric and body composition variables, the BMI and waist circumference best explained the EAT-26 variance [24], possibly explaining the fact that, in the present study, EAT-26 was not related to the body composition variables. However, most studies relied on simple variable correlations instead of multivariate models. Anxiety had a negative association with BMI (simple correlation), as previously noted [18].

In comparison to prior research, this study presents several strengths. Anthropometry and body composition measurements were performed at the University. Unlike other studies, [5,15,16,20,23,25,26,56], the data on body weight were not self-reported. Validated questionnaires, such as EAT-26, which have been extensively used in student populations as screening tools, were also employed.

However, there are several limitations to consider. The current study is cross-sectional. As such, it is unable to establish etiological relationships between factors. In addition, the sample used solely comprised students from one university, which may limit the generalizability of the findings to the wider population. Regarding the EAT-26 and DASS questionnaires, it is crucial to note that they do not function as diagnostic tools for eating disorders or for depression and anxiety disorders [30,65].

## 5. Conclusions

In conclusion, it was shown that a proportion of students at the specific university are at an increased risk of developing eating disorders, while they may experience depression, anxiety, and stress. The presence of disordered eating was positively associated with the presence of psychological symptoms. In addition, depression, anxiety, and stress were found to be significantly associated with the students’ body weight status. The risk of developing eating disorders was associated with a higher BMI, while depression was associated with a lower BMI. Our results underline the need to create appropriate learning environments for Dietetics students to openly discuss their struggles and enhance their well-being. Moreover, further research is needed to explore eating attitudes and depression, anxiety, and stress in Dietetics professionals.

## Figures and Tables

**Table 1:** Descriptive characteristics of respondents based on their answers on EAT-26.

	Total	EAT-26 < 20	EAT-26 > 20	*p*-Value
Number of respondents (n)	143	120	23	
Sex				0.468
*Female (n)*	108	92	16	
*Male (n)*	35	28	7	
Age (years)	20.95 ± 4.20	21.04 ± 4.47	20.5 ± 2.20	0.391
Year of studies				0.354
*1st (n)*	45	35	9	
*2nd (n)*	47	32	8	
*3rd (n)*	44	40	4	
*4th (n)*	12	11	1	
BMI (kg/m^2^)	22.07 ± 2.94	22.02 ± 2.84	22.35 ± 3.50	0.672
*Underweight (n)*	15	11	3	
*Normal weight (n)*	115	92	16	
*Overweight and Obese (n)*	21	17	4	
Fat mass (%)	22.97 ± 7.68	23.44 ± 7.39	20.63 ± 8.87	0.165
Fat mass (kg)	14.21 ± 5.67	14.47 ± 5.49	12.90 ± 6.53	0.290
Wrist circumference (cm)	15.50 (14.75–16.25)	15.5 (15.0–16.3)	15.35 (14.5–16.1)	0.645
Waist circumference (cm)	71.00 (67.00–75.50)	71.0 (67.0–75.4)	70.5 (67.8–77.0)	0.876
Hip circumference (cm)	97.00 (93.00–101.00)	97.0 (93.0–101.0)	94.0 (91.7–101.5)	0.552
Biceps skinfold (mm)	8.00 (5.23–12.40)	8.6 (5.5–12.5)	5.9 (4.2–12.0)	0.119
Triceps skinfold (mm)	14.60 (11.80–20.00)	15.0 (12.0–20.3)	13.2 (9.4–15.7)	**0.029**
Subscapular skinfold (mm)	11.30 (9.55–15.3)	11.5 (9.6–15.3)	10.6 (8.5–15.0)	0.438
Suprailiac skinfold (mm)	10.30 (7.91–13.50)	10.6 (8.3–14.0)	8.3 (5.5–11.0)	**0.008**
Sum of skinfolds (mm)	46.6 (37.1–60.9)	46.8 (38.5–61.5)	36.9 (30.2–52.0)	**0.027**
Physical activity				
*Total MET/minutes*	1832 (1689–2001)	1822 (1695–1994)	1881 (1668–2059)	0.811
*Sleep duration (hours)*	7.00 (6.00–8.00)	7.3 (7.0–8.0)	7.0 (6.0–8.0)	0.094

Values represent means ± standard deviations (for variables following a normal distribution) or medians and 25th–75th percentiles (for variables not following a normal distribution). Categorical variables are displayed as frequencies. *T*-test (for normal variables) or Mann–Whitney test (for non-normal variables) was used to compare values between men and women. For comparisons between categorical variables, the chi-square test was used. Bold denotes statistically significant differences.

**Table 2:** Spearman correlations between EAT-26 and DASS.

		DASS Total		DASS Depression		DASS Anxiety		DASS Stress	
		Correlation Coefficient	*p*-Value	Correlation Coefficient	*p*-Value	Correlation Coefficient	*p*-Value	Correlation Coefficient	*p*-Value
**Total**	EAT-26 Total	**0** **.** **221**	**0** **.** **010**	0.146	0.090	**0.** **202**	**0** **.** **017**	**0.** **282**	**0** **.** **001**
	EAT-26 Dieting	**0.** **214**	**0** **.** **013**	0.156	0.068	**0.** **209**	**0** **.** **013**	**0.** **250**	**0** **.** **003**
	EAT-26 Bulimia	**0.** **217**	**0** **.** **011**	0.112	0.191	**0.** **167**	**0** **.** **047**	**0.** **279**	**0** **.** **001**
	EAT-26 Oral Control	**0.** **177**	**0** **.** **039**	**0.** **179**	**0** **.** **035**	**0.** **177**	**0** **.** **034**	**0.** **204**	**0** **.** **015**
**Women**	EAT-26 Total	**0.** **280**	**0.** **004**	**0** **.** **224**	**0** **.** **023**	**0.** **259**	**0** **.** **007**	**0.** **320**	**0** **.** **001**
	EAT-26 Dieting	**0.** **255**	**0** **.** **009**	**0.** **265**	**0** **.** **006**	**0.** **237**	**0** **.** **014**	**0.** **275**	**0** **.** **004**
	EAT-26 Bulimia	**0.** **271**	**0** **.** **005**	0.138	0.156	**0.** **208**	**0** **.** **030**	**0.** **319**	**0** **.** **001**
	EAT-26 Oral Control	**0** **.** **188**	**0** **.** **053**	0.176	0.069	**0.** **219**	**0** **.** **022**	**0** **.** **188**	**0** **.** **050**
**Men**	EAT-26 Total	0.070	0.709	−0.105	0.568	0.074	0.684	0.197	0.271
	EAT-26 Dieting	0.098	0.599	−0.149	0.415	0.160	0.374	0.190	0.291
	EAT-26 Bulimia	0.134	0.472	0.050	0.786	0.061	0.738	0.198	0.270
	EAT-26 Oral Control	0.213	0.250	0.124	0.500	0.072	0.690	0.327	0.064

Bold denotes statistically significant differences.

**Table 3:** Linear regression analyses with BMI as dependent variable and EAT-26 or DASS scores as independent variables in men.

Independent Variable (in All Models): Body Mass Index (kg/m^2^)	Model 1: Age (Years)		Model 2: Model 1 + Physical Activity (Total MET/min)		Model 3: Model 2 + Mediterranean Dietary Score (0–55)	
Dependent Variables	b (SE)	*p*-Value	b (SE)	*p*-Value	b (SE)	*p*-Value
EAT-26 Total	0.088 (0.053)	0.105	0.094 (0.050)	0.069	0.091 (0.053)	0.095
EAT-26 Dieting	0.069 (0.115)	0.553	0.081 (0.117)	0.492	0.079 (0.121)	0.520
EAT-26 Bulimia	0.284 (0.189)	0.143	0.271 (0.182)	0.146	0.259 (0.200)	0.204
**EAT-26 Oral Control**	**0.440 (0.187)**	**0.025**	**0.441 (0.178)**	**0.019**	**0.430 (0.184)**	**0.026**
DASS Total	−0.038 (0.026)	0.160	−0.042 (0.025)	0.111	−0.042 (0.026)	0.117
DASS Depression	−0.148 (0.077)	0.064	−0.145 (0.075)	0.064	−0.141 (0.076)	0.076
DASS Anxiety	−0.119 (0.085)	0.173	−0.137 (0.082)	0.107	−0.136 (0.083)	0.114
DASS Stress	−0.058 (0.055)	0.300	−0.071 (0.054)	0.197	−0.072 (0.054)	0.193

SE: standard error; bold denotes statistically significant differences.

## Data Availability

The data are available upon request.

## Supplementary Materials

The following supporting information can be downloaded at: https://www.mdpi.com/article/10.3390/diseases12050108/s1, Table S1: Descriptive characteristics of respondents based on their answers on DASS; Table S2: Spearman correlations between EAT-26 and anthropometric/ body composition variables in the whole sample; Table S3: Spearman correlations between EAT-26 and anthropometric/body composition variables in women; Table S4: Spearman correlations between EAT-26 and anthropometric/ body composition variables in men; Table S5: Linear regression analyses with BMI as dependent variable and EAT-26 as independent variable in women.
