# Supplementary material for: Eating Attitudes, Stress, Anxiety, and Depression in Dietetic Students and Association with Body Mass Index and Body Fat Percent: A Cross-Sectional Study
Source: Diseases. 2024 May 20;12(5):108. doi: 10.3390/diseases12050108 (PMC11120143; doi:10.3390/diseases12050108)
Supplement: Supplementary file 1 [file diseases-12-00108-s001.zip › diseases-2954800-supplementary.pdf]

Eating Attitudes, Stress, Anxiety, and Depression in Dietetic Students and Association with Body Mass Index and Body Fat Percent: A Cross-Sectional Study

Electronic Supplementary Material

Supplementary Table S1. Descriptive characteristics of respondents based on their answers on DASS.

	Total	DASS score: 0-9	DASS score: 10-12	DASS score: 13-20	DASS score: 21-27	DASS score: ≥28	p-value
Number of participants (n)	139	30	12	29	21	47	
Sex							0.382
Female (n)	107	20	8	25	17	37	
Male (n)	32	10	4	4	4	10	
Age (years)	20. 97 ±4.32	22.1 ±6.4	20.5 ±1.6	21.1 ±5.0	20.9 ±4.1	20.4 ±2.2	0.626
Year of studies							0.243
1st (n)	43	8	1	11	7	16	
2nd (n)	39	6	8	7	7	11	
3rd (n)	42	11	2	9	6	14	
4th (n)	12	5	1	1	1	4	
BMI (kg/m^2)	22.14 ±2.92	22.9 ±3.5	22.8 ±2.9	22.5 ±2.9	21.4 ±2.2	21.5 ±2.6	0.147
Underweight (n)	12	3	1	2	1	5	
Normal weight (n)	107	19	10	20	19	39	
Overweight and Obese (n)	20	8	1	7	1	3	
Fat mass (%)	23.07 ±7.50	23.2 ±8.0	23.9 ±9.0	24.0±6.0	22.2±7.9	22.6±7.3	0.912
Fat mass (kg)	14.27 ±5.44	15.2±6.4	15.3±6.3	14.7±4.7	13.3±5.1	13.6±4.9	0.577
Wrist Circumference (cm)	15.5 (15.0-16.3)	15.6 (14.5-16.5)	15.8 (15.0-16.8)	16.0 (15.4-16.0)	15.3 (14.8-16.1)	15.0 (14.5-16.5)	0.517
Waist Circumference (cm)	70.7 (67.0–75.0)	72.0 (68.6-78.3)	72.0 (65.9-77.9)	70.0 (65.0-77.3)	70.5 (66.5-75.0)	70.0 (65.3-75.0)	0.237
Hip Circumference (cm)	97.0 (93.0–101.0)	99.3 (92.8-105.3)	98.8 (96.3-101.8)	97.0 (93.0-101.0)	97.0 (92.0-99.0)	95.8 (92.6-99.0)	0.262
Biceps Skinfold (mm)	8.6 (5.3–12.8)	7.5 (5.0-11.5)	8.8 (4.1-15.8)	9.5 (7.2-15.8)	8.5 (5.6-8.5)	7.4 (5.0-11.4)	0.147

Triceps Skinfold (mm)	14.6 (12.0–19.5)	15.3 (12.2-17.3)	16.4 (9.0-22.7)	15.6 (13.9-21.3)	14.3 (11.4-20.1)	13.9 (11.4-16.5)	0.371
Subscapular Skinfold (mm)	11.50 (9.60–15.30)	12.2 (9.2-17.3)	12.4 (10.2-18.3)	11.3 (10.0-15.3)	12.0 (8.3-15.5)	10.7 (9.4-13.8)	0.264
Suprailiac Skinfold (mm)	10.30 (8.00–13.50)	12.5 (8.3-14.1)	9.8 (7.7-13.6)	11.3 (9.2-15.1)	10.0 (7.1-14.0)	10.1 (8.0-12.2)	0.450
Sum of Skinfolds (mm)	46.6 (37.1–60.9)	51.0 (36.9-63.1)	45.2 (31.7-70.2)	48.0 (40.7-69.9)	44.5 (34.4-59.4)	42.1 (35.2-52.9)	0.236
Physical Activity							
Total MET/minutes	1842 (1705–2037)	1827 (1743-2080)	1743 (1653-1925)	1872 (1685-1979)	1808 (1679-2035)	1848 (1758-2173)	0.460
Sleep duration (hours)	7.00 (6.87-8.00)	7.0 (6.0-8.0)	7.0 (7.0-8.0)	8.0 (7.0-8.0)	8.0 (7.0-8.0)	7.0 (6.0-8.0)	0.650

Values represent means ± standard deviations (for normally distributed variables) or medians and interquartile ranges (for non-normally distributed variables). Categorical variables are displayed as frequencies. T-test (for normal variables) or Mann–Whitney test (for non-normal variables) was used to compare values between men and women. For comparisons between categorical variables the chi-square test was used.

Supplementary Table S2. Spearman correlations between EAT-26 and anthropometric/ body composition variables in the whole sample

		EAT-26 Total		EAT-26 Dieting		EAT-26 Bulimia		EAT-26 Oral Control	
		Correlation coefficient	p-value	Correlation coefficient	p-value	Correlation coefficient	p-value	Correlation coefficient	p-value
**Total**	Age (years)	0.024	0.773	-0.037	0.662	-0.055	0.514	0.136	0.101
	BMI (kg/m^2)	0.037	0.663	**-0.162**	**0.050**	0.158	0.057	**0.178**	**0.031**
	Fat mass (%)	-0.002	0.977	-0.049	0.560	0.048	0.569	0.018	0.828
	Fat mass (kg)	-0.061	0.472	**-0.162**	**0.053**	0.040	0.639	0.018	0.826
	Phase Angle (°)	0.050	0.557	-0.039	0.643	0.077	0.359	0.072	0.391
	Wrist Circumference (cm)	-0.114	0.180	**-0.180**	**0.031**	<0.001	0.996	-0.040	0.633
	Waist Circumference (cm)	-0.036	0.677	**-0.235**	**0.005**	0.079	0.351	0.142	0.090
	Hip Circumference (cm)	-0.081	0.341	**-0.187**	**0.025**	0.035	0.678	0.004	0.961
	Biceps Skinfold (mm)	-0.044	0.608	-0.046	0.589	-0.020	0.813	-0.046	0.586
	Triceps Skinfold (mm)	**-0.172**	**0.042**	**-0.220**	**0.008**	-0.057	0.496	-0.087	0.229
	Subscapular Skinfold (mm)	-0.079	0.353	**-0.191**	**0.022**	0.012	0.886	0.013	0.878
	Supra iliac Skinfold (mm)	-0.072	0.397	-0.094	0.262	-0.065	0.441	0.027	0.746
	Sum of Skinfolds (mm)	-0.117	0.167	**-0.174**	**0.038**	-0.040	0.631	-0.034	0.686
	Physical Activity								
	Total MET/minutes/week	0.027	0.747	-0.024	0.770	0.068	0.415	0.073	0.379
	Sleep duration (hours)	-0.121	0.149	-0.036	0.662	-0.159	0.056	-0.115	0.167

Bold denotes statistically significant differences.

Supplementary Table S3. Spearman correlations between EAT-26 and anthropometric/body composition variables in women

		EAT-26 Total		EAT-26 Dieting		EAT-26 Bulimia		EAT-26 Oral Control	
		Correlation coefficient	p-value	Correlation coefficient	p-value	Correlation coefficient	p-value	Correlation coefficient	p-value
**Women**	Age (years)	0.060	0.542	0.021	0.825	-0.071	0.459	0.135	0.157
	BMI (kg/m^2)	0.017	0.865	-0.153	0.111	0.147	0.125	0.108	0.259
	Fat mass (%)	0.013	0.895	-0.093	0.342	0.113	0.243	0.057	0.553
	Fat mass (kg)	-0.044	0.653	-0.170	0.081	0.097	0.316	0.027	0.780
	Phase Angle (º)	0.037	0.706	-0.024	0.803	0.021	0.826	0.060	0.532
	Wrist Circumference (cm)	**-0.191**	**0.050**	**-0.232**	**0.016**	-0.060	0.536	-0.103	0.284
	Waist Circumference (cm)	-0.047	0.634	**-0.228**	**0.018**	0.044	0.651	0.109	0.256
	Hip Circumference (cm)	-0.158	0.106	**-0.238**	**0.014**	-0.021	0.832	-0.106	0.272
	Biceps Skinfold (mm)	-0.003	0.976	-0.079	0.420	0.095	0.326	0.018	0.851
	Triceps Skinfold (mm)	-0.149	0.128	**-0.254**	**0.008**	0.043	0.655	-0.063	0.514
	Subscapular Skinfold (mm)	-0.087	0.377	**-0.188**	**0.052**	0.016	0.868	<0,001	0.998
	Supra iliac Skinfold (mm)	0.054	0.582	-0.001	0.989	0.025	0.800	0.144	0.134
	Sum of Skinfolds (mm)	-0.062	0.528	-0.168	0.083	0.060	0.540	0.021	0.827
	Physical Activity								
	Total MET/minutes/week	0.079	0.418	0.016	0.867	0.093	0.332	0.067	0.482
	Sleep duration (hours)	-0.138	0.154	-0.064	0.506	**-0.216**	**0.023**	-0.099	0.301

Bold denotes statistically significant differences.

Supplementary Table S4. Spearman correlations between EAT-26 and anthropometric/ body composition variables in men

		EAT-26 Total		EAT-26 Dieting		EAT-26 Bulimia		EAT-26 Oral Control	
		Correlation coefficient	p-value	Correlation coefficient	p-value	Correlation coefficient	p-value	Correlation coefficient	p-value
**Men**	Age (years)	-0.128	0.465	-0.219	0.200	-0.018	0.920	0.163	0.350
	BMI (kg/m^2)	0.029	0.868	-0.175	0.306	0.106	0.544	0.304	0.076
	Fat mass (%)	-0.152	0.384	**-0.347**	**0.038**	-0.025	0.889	0.045	0.799
	Fat mass (kg)	-0.091	0.603	**-0.329**	**0.050**	0.035	0.841	0.108	0.535
	Phase Angle (°)	0.109	0.534	0.040	0.818	0.268	0.119	0.098	0.574
	Wrist Circumference (cm)	-0.018	0.919	0.050	0.771	0.171	0.325	-0.071	0.684
	Waist Circumference (cm)	-0.205	0.253	-0.299	0.086	0.004	0.983	0.034	0.852
	Hip Circumference (cm)	0.130	0.455	-0.024	0.892	0.222	0.199	0.311	0.069
	Biceps Skinfold (mm)	-0.143	0.411	-0.172	0.315	-0.146	0.402	0.013	0.942
	Triceps Skinfold (mm)	-0.282	100	**-0.361**	**0.031**	-0.265	0.124	0.023	0.896
	Subscapular Skinfold (mm)	-0.035	0.840	-0.211	0.216	0.008	0.965	0.070	0.689
	Supra iliac Skinfold (mm)	**-0.442**	**0.008**	**-0.389**	**0.019**	**-0.349**	**0.040**	-0.269	0.118
	Sum of Skinfolds (mm)	-0.274	0.111	**-0.327**	**0.051**	-0.246	0.154	-0.024	0.893
	Physical Activity								
	Total MET/minutes/week	-0.106	0.544	-0.158	0.357	0.003	0.985	0.096	0.585
	Sleep duration (hours)	-0.067	0.700	0.059	0.733	0.003	0.984	-0.194	0.264

Bold denotes statistically significant differences.

Supplementary Table S5. Linear regression analyses with BMI as dependent variable and EAT-26 as independent variable in women.

Independent Variable (in all models): BMI	Model 1: Age (years)		Model 2: Model 1 + Physical Activity (Total MET/minute/day)		Model 3: Model 2 + Mediterranean Dietary Score (0-55)	
**Dependent Variables**	**b (SE)**	**p-value**	**b (SE)**	**p-value**	**b (SE)**	**p-value**
EAT-26 Total	-0.006 (0.040)	0.876	-0.006 (0.040)	0.887	-0.010 (0.041)	0.808
EAT-26 Dieting	-0.104 (0.070)	0.142	-0.103 (0.070)	0.148	-0.112 (0.071)	0.120
EAT-26 Bulimia	0.208 (0.142)	0.148	0.213 (0.144)	0.142	0.226 (0.148)	0.131
EAT-26 Oral Control	0.030 (0.111)	0.786	0.030 (0.111)	0.787	0.027 (0.113)	0.815
DASS Total	-0.011 (0.015)	0.457	-0.011 (0.015)	0.473	-0.009 (0.015)	0.566
DASS Depression	-0.013 (0.040)	0.750	-0.013 (0.041)	0.749	-0.008 (0.041)	0.847
DASS Anxiety	-0.059 (0.043)	0.173	-0.059 (0.043)	0.175	-0.050 (0.044)	0.256
DASS Stress	-0.025 (0.034)	0.467	-0.025 (0.035)	0.466	-0.023 (0.035)	0.506

BMI: Body Mass Index (kg/m2), SE: Standard Error
